# Carbon availability in *Staphylococcus aureus* infection: cellular access, metabolic fate and antibiotic response

**DOI:** 10.1007/s11274-026-05408-9

**Published:** 2026-11-13

**Authors:** Rafaella Coelho Oliveira, Vitor da Silva Moraes, João Inácio Diniz Ferreira, Weldson Ricardo Silva Gomes, Camila Guerra Martinez

**Affiliations:** Universidade CEUMA, São Luís, Maranhão Brazil

**Keywords:** Antibiotic tolerance, Antimicrobial resistance, Antibiotic killing, Carbon metabolism, Host–pathogen interactions, Physiological heterogeneity

## Abstract

Carbon availability during *Staphylococcus aureus* infection is often equated with extracellular substrate concentration, although this measurement reveals neither cellular partitioning nor the intracellular fate of acquired carbon. This distinction becomes treatment-relevant because conditions that permit bacterial survival need not be those that later favor population recovery or resistant genotypes. Here, we synthesize infection-relevant evidence across five inferential levels: local substrate pool, cellular access, intracellular carbon fate, physiological state and antibiotic response. Applying this separation across time distinguishes immediate metabolic effects on killing from subsequent population recovery and resistant-variant expansion. In a diabetic skin-infection model, hyperglycaemia increased recovery of heritable rifampicin resistance without genomic evidence of hypermutability, consistent with altered population opportunity rather than a direct carbon-to-mutation mechanism. Across the evidence reviewed, no study in *S. aureus* has linked the measured carbon fate of a bacterium surviving antibiotic exposure to the recovery and resistance phenotypes of its descendants. We therefore propose a two-stage, non-obligatory model in which restrictive carbon conditions can modify survival during treatment, whereas restoration or rerouting of metabolically usable carbon can restore killing, permit susceptible regrowth or favor expansion of resistant variants. The model yields a lineage-specific, falsifiable prediction: carbon-restricted survivors contribute disproportionately to the recovered population and, under continued selection, to its resistant fraction. Testing this prediction requires experiments that preserve substrate identity, cellular identity, intracellular carbon fate and ancestry through recovery. By defining this unconnected causal step, the framework makes cellular descent testable and provides a basis for evaluating metabolic interventions across antibiotic–niche combinations.

## Introduction

Carbon metabolism shapes *Staphylococcus aureus* infection not only by supplying energy and biosynthetic precursors, but also by determining whether bacterial cells can maintain respiration, redox balance, envelope synthesis and other functions required for antibiotic action. Expanded glucose transport, for example, supports glycolytic growth when respiration is limited during tissue invasion (Vitko et al. 2016). During treatment, the question is which cell obtains the substrate, how the bacterium routes it, what physiological state results and which population is subsequently recovered. An extracellular concentration cannot by itself resolve any of those transitions.

Host and bacterial carbon metabolism operate within the same infected microenvironment. Host cells alter nutrient transport and consumption while mounting antimicrobial responses, whereas *S. aureus* can change substrate use as oxygen availability, immune pressure, tissue composition and infection duration change. Epithelial glucose transport can restrict glucose accumulation at mucosal surfaces (Garnett et al. 2014), myeloid glucose uptake can sustain antimicrobial NADPH production (Banerjee et al. 2024), and bacterial glucose transport can support glycolytic growth during infection (Vitko et al. 2016). The resulting carbon environment reflects cellular uptake, metabolic conversion and reciprocal host–pathogen remodeling; extracellular concentration captures only part of this process.

Antibiotic tolerance describes slower population killing without an increase in minimum inhibitory concentration (MIC). Persistence describes tolerance expressed by a surviving subpopulation, typically detected as biphasic killing (Fisher et al. 2017; Brauner et al. 2016; Balaban et al. 2019). Resistance is the heritable capacity to grow at antibiotic concentrations that inhibit the susceptible population; its fitness costs can vary with the environment (Andersson and Hughes 2010; Brauner et al. 2016). These labels do not imply a single underlying mechanism. We retain the term persister only when supported by the experimental design or used operationally by the cited study, rather than inferring persistence from survival at a single time point.

Existing reviews answer adjacent but different questions. Nutrient-based potentiation of antibiotic activity and pathogen adaptation to lung metabolites have been examined in recent reviews (Peng et al. 2025; Lohia and Riquelme 2025). Other syntheses address metabolic rewiring as a response to antibiotic pressure across pathogens or the energetic barrier to aminoglycoside uptake in *S. aureus* persisters (Acierno et al. 2025; Baek et al. 2026). Together, these perspectives explain how metabolic state can modify drug activity or be therapeutically reprogrammed. They do not organize the infection evidence around the causal continuity from a host-accessible substrate pool to cellular partitioning, bacterial carbon fate, antibiotic survival and the ancestry of the population recovered after treatment. We address this continuity across the stages of infection and treatment. This separation is also necessary for intervention design, because increasing or restricting bacterial carbon use cannot be interpreted as intrinsically beneficial: its effect depends on the physiological process altered, the antibiotic applied and the subsequent opportunity for population recovery.

That distinction matters during antimicrobial treatment. Host-induced respiratory disruption can reduce bacterial ATP and increase antibiotic tolerance without requiring extracellular carbon depletion (Rowe et al. 2020), whereas provision of metabolically usable glucose can increase daptomycin killing of stationary-phase persisters (Prax et al. 2016). Host-derived triglycerides can instead support biofilm-associated tolerance through Geh-dependent glycerol release and wall-teichoic-acid remodeling (Ledger et al. 2026). Carbon availability cannot be assigned a fixed relationship with antibiotic efficacy from substrate concentration alone.

Population consequences add a further level of uncertainty. In diabetic skin infection, hyperglycaemia increases *S. aureus* burden and recovery of rifampicin-resistant populations without genomic evidence of hypermutability (Shook et al. 2025). The remaining question is how observations made at these causal levels connect across time. No current *S. aureus* study follows a defined host-accessible substrate through cellular partitioning and bacterial metabolic fate, identifies the cells that survive treatment in the resulting physiological state, and determines whether their descendants dominate the recovered or resistant population. We address this gap by treating carbon availability as a property of the host–pathogen system, separating immediate killing from post-treatment population dynamics, and converting the proposed connection between them into a lineage-specific prediction with explicit criteria for rejection.

This is a mechanistic narrative review rather than a systematic inventory. Primary studies were included when they experimentally resolved at least one link among extracellular substrate availability, cellular acquisition, intracellular carbon fate, bacterial physiological state, antibiotic killing or the fitness of resistant populations. Studies limited to descriptive metabolite abundance were not used to infer cellular acquisition or carbon fate, and survival on antibiotic-containing medium was not treated as heritable resistance without post-isolation susceptibility evidence. Recent reviews were used to delimit adjacent conceptual and therapeutic fields. Evidence from other bacterial species was included only when it established an experimental or evolutionary boundary that remains unresolved in *S. aureus*, and is identified as such where discussed. The evidence base was delimited by mechanistic relevance rather than publication date. Recent infection studies were prioritized, whereas earlier work was retained when it established experimental definitions, recovery kinetics or evolutionary precedents required to interpret the current literature.

**Fig. 1:**
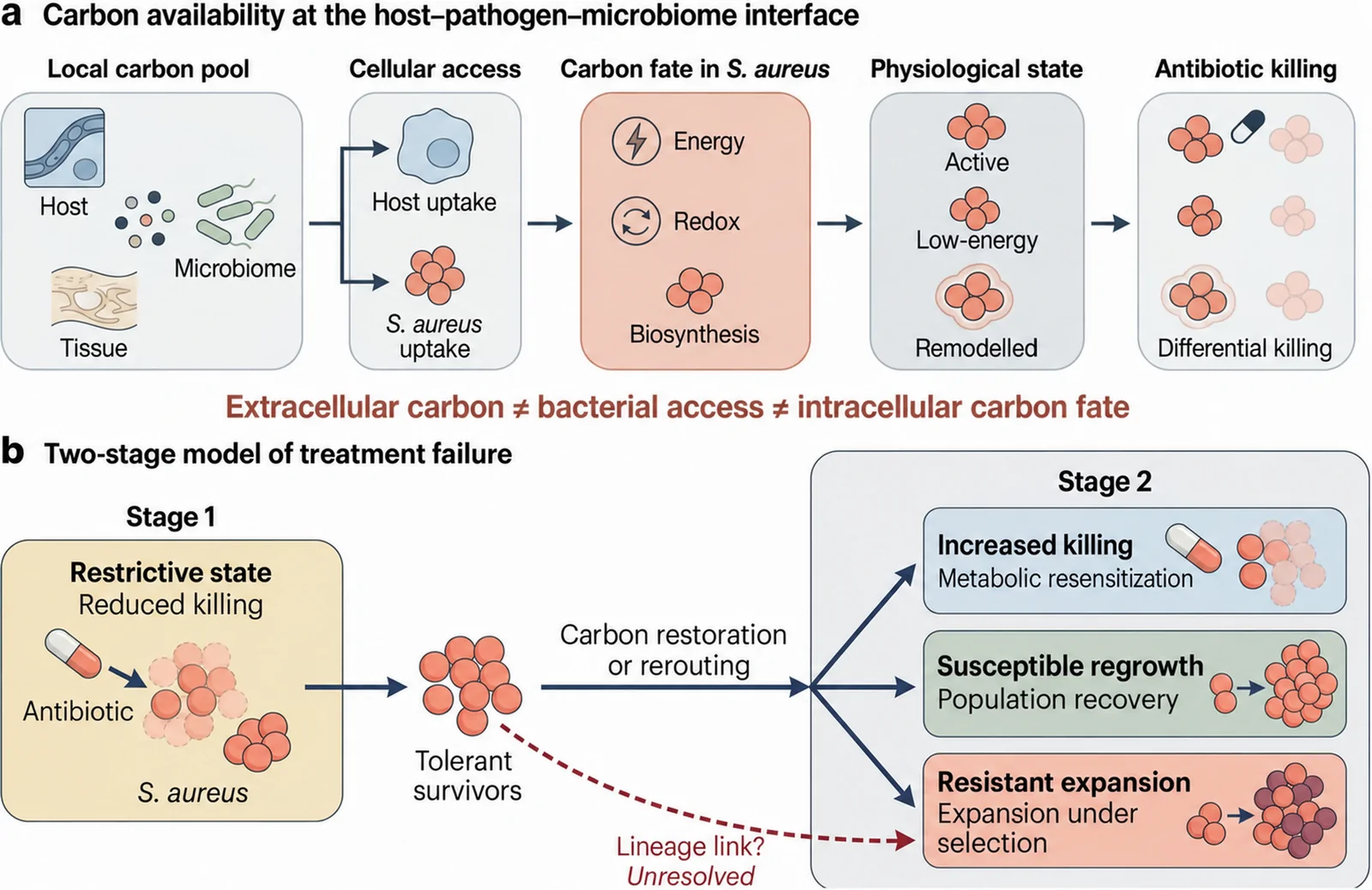
Carbon availability at the host–pathogen–microbiome interface and its proposed relationship to treatment failure. (**a**) Local carbon pools are jointly shaped by host transport and consumption, microbiome competition and metabolite production, and *Staphylococcus aureus* acquisition and intracellular routing. Extracellular concentration therefore does not directly specify bacterial cellular access or intracellular carbon fate. The left-to-right organization separates analytical levels; the underlying relations are interdependent and need not form a strictly linear sequence. (**b**) In the two-stage model, a restrictive condition can reduce antibiotic killing when it produces a low-energy or otherwise tolerant physiological state for a defined antibiotic–niche combination. Subsequent restoration or rerouting of metabolically usable carbon can increase killing through metabolic resensitization, permit susceptible regrowth, or support expansion under conditions that favor resistant cells. These outcomes are not mutually exclusive at the population level, and none constitutes an obligatory progression. Solid arrows denote mechanistic or temporal relations supported within defined experimental settings. The dotted arrow marks the unresolved lineage hypothesis: whether Stage 1 survivors disproportionately seed the resistant population recovered in Stage 2

## Carbon availability emerges from reciprocal metabolic control at the host–pathogen interface

Carbon sources encountered by *S. aureus* during infection are determined by more than their extracellular concentrations. Transport across tissue barriers, uptake by host and bacterial cells, metabolic conversion after uptake, and release of substrates during tissue remodeling all influence whether a carbon source can support bacterial metabolism. These processes can also change during infection as inflammatory responses alter host metabolism and bacterial populations adapt to the nutritional conditions imposed by the infected tissue.

Glucose illustrates this problem because it is simultaneously a bacterial substrate and a host metabolic resource. *S. aureus* can use glucose to support central carbon metabolism, ATP production and virulence-associated functions, whereas immune cells use glucose-derived reducing power to sustain antimicrobial activity. The concentration measured in an infected compartment represents the substrate remaining after processes that can have opposing consequences for bacterial survival. Determining what that concentration means requires resolving which cells acquire the available carbon, what metabolic pathways process it, and whether those capacities remain stable as infection progresses.

### Extracellular carbon pools do not define cellular access

At epithelial surfaces, the carbon pool encountered by *S. aureus* is constrained by host transport. Polarized glucose uptake, intracellular phosphorylation and low paracellular permeability maintain low airway-surface glucose in differentiated human epithelial models (Pezzulo et al. 2011). The infection experiments in that study used *Pseudomonas aeruginosa*, providing a barrier-physiology precedent rather than a direct *S. aureus* model. In an airway epithelial model, increasing basolateral sugar concentration increases apical bacterial growth, while epithelial glucose uptake limits glucose accumulation in airway surface liquid and thereby restricts sugar-supported bacterial growth (Garnett et al. 2014). Reducing airway glucose permeability with metformin also lowers hyperglycaemia-associated *S. aureus* burden independently of systemic glucose lowering (Garnett et al. 2013). The substrate concentration supplied to the basolateral compartment does not directly specify the carbon pool encountered by bacteria at the epithelial surface. Host metabolism can also alter the fraction of a local pool available to bacteria. In primary human epidermal keratinocytes infected with USA300 LAC, infection increases host glycolytic activity, whereas inhibition of host glycolysis impairs protective inflammatory responses and worsens dermonecrosis in vivo (Wickersham et al. 2017). These experiments demonstrate host control over carbon availability without quantifying carbon partition between host and bacterial cells.

Diabetic skin infection exposes a different allocation problem. Streptozotocin-induced hyperglycaemia increases glucose within infected tissue and is accompanied by larger lesions, greater bacterial burden and dissemination (Thurlow et al. 2020). Phagocytes recovered from hyperglycaemic infection express less GLUT1 and GLUT3, and glucose deprivation impairs macrophage oxidative burst and bacterial killing. In the same model, bacterial defects in glucose transport or glucose-to-glucose-6-phosphate processing reduce ATP production and virulence-associated phenotypes (Thurlow et al. 2020). Increased tissue glucose coincides with greater opportunity for bacterial glucose utilization and impaired host capacity to convert glucose into antimicrobial activity.

The bacterial advantage associated with hyperglycaemia cannot be attributed to extracellular glucose concentration alone. An expanded tissue pool can increase substrate accessible to *S. aureus* while reduced host uptake weakens a glucose-dependent defense operating in the same compartment. Measuring the pool without resolving its acquisition cannot distinguish these mechanisms.

### Carbon acquisition does not determine intracellular fate

Low extracellular glucose does not imply global carbon starvation because *S. aureus* can redirect metabolism toward alternative substrates. Amino acid catabolism supports central metabolism when preferred carbon sources are limited, and carbon catabolite repression determines which amino acids contribute substantially under different nutritional conditions (Halsey et al. 2017). Oxygen availability further changes the fate of acquired carbon: glucose metabolism and lactate utilization differ between aerobic and oxygen-limited conditions, and anaerobic growth produces carbon-source-dependent reprogramming of central metabolism (Ferreira et al. 2013; Troitzsch et al. 2021). Which substrate can substitute for glucose depends on the niche: local substrate delivery and competition determine access, whereas oxygen tension, transport capacity and the integrity of downstream pathways determine whether acquisition yields ATP, reducing power or biosynthetic precursors.

Carbon fate also depends on the metabolic routes that remain functional after uptake. Expanded glucose transport capacity allows *S. aureus* to maximize glucose uptake and supports glycolytic growth under respiration-limiting conditions encountered during infection (Vitko et al. 2016). The physiological consequence of that acquisition is determined by the metabolic routes available after uptake and by the respiratory conditions in which the acquired carbon is processed. Recent infection models reinforce this separation between extracellular substrate and intracellular use. Host-niche conditions alter which substrates support *S. aureus* survival, and fumarate availability can be coupled to distinct downstream biosynthetic and energetic routes rather than functioning as a simple extracellular abundance signal (Chang et al. 2025; Chen et al. 2025).

Intracellular carbon fate can also provide regulatory information rather than only energy or material flux. In *S. aureus*, intracellular citrate activates CcpE, alters expression of genes involved in central carbon metabolism, iron uptake and virulence, and suppresses Fur regulatory activity; citrate accumulation, partly through CcpE, also decreases pathogenic potential in animal infection models (Chen et al. 2024). CcpA and CodY provide a broader nutrient-responsive architecture: single and double regulatory mutants in UAMS-1 alter carbon flux through glycolytic, overflow, TCA-cycle and amino-acid pathways and produce distinct virulence and biofilm phenotypes (Bulock et al. 2022). Thus, the consequence of acquired or internally generated carbon depends not only on pathway capacity but also on how metabolic state is interpreted by regulatory networks.

The physiological consequences of glucose availability depend on the metabolic routes that remain accessible after uptake or depletion. Low extracellular glucose can coexist with continued ATP generation from alternative substrates, whereas abundant glucose cannot sustain the same output when downstream processing is constrained. Extracellular concentration and substrate uptake alone cannot resolve which of these states the bacterium occupies.

### Carbon use changes across anatomical transitions

The transition from a colonized or infected tissue into the bloodstream exposes *S. aureus* to new carbon and stress conditions. In five clinically relevant lineages, integrated transcriptomic, proteomic and metabolomic analyses after human-serum exposure identified coordinated changes in carbon metabolism and stress responses (Mujchariyakul et al. 2026). Gluconeogenic and TCA-cycle activity increased alongside broader use of carbohydrate, amino-acid and lipid substrates. Iron-acquisition systems and oxidative-stress defenses were induced, whereas ribosome biogenesis was suppressed (Mujchariyakul et al. 2026). Competitive assays with USA300 JE2 transposon mutants supported roles for the carbon-metabolism genes *gapdhB* and *sucA* in serum fitness, alongside iron-transport and peroxide-response functions. Serum survival thus depends on coordinated carbon use and stress adaptation, not on a single limiting substrate.

Whole blood and serum adaptation resolve complementary parts of this transition. *S. aureus* can use glucose and lactate simultaneously in human blood, unlike their sequential utilization in laboratory medium (Chang et al. 2025). Serum adaptation also increases daptomycin tolerance through LL-37–GraRS-dependent peptidoglycan accumulation and a separate cardiolipin response (Ledger et al. 2022). These findings connect the blood environment with substrate use and treatment response, while showing that serum exposure changes more than carbon supply.

These data sharpen, but do not close, the anatomical transition problem. Serum is an acellular proxy for one component of blood and does not reproduce blood cells, endothelium, source-tissue metabolites or the history of the disseminating population (Mujchariyakul et al. 2026; Rose and Sauer 2026). These experiments do not track bacteria as they leave an infection focus and enter blood. Marked *S. aureus* populations already reveal dissemination bottlenecks and clonal expansion in infection models, providing a precedent for retaining source-population identity (Pollitt et al. 2018). Resolving that transition will require paired or temporally linked models that retain the identity of the source population while measuring how carbon flux, iron acquisition and antibiotic susceptibility change during dissemination.

### Bacterial adaptation changes the fitness value of host carbon sources

The metabolic value of a host-derived substrate can change as infection progresses. Environmental cues in different host niches alter the contribution of specific metabolic functions to *S. aureus* survival, demonstrating that substrate use is conditioned by the local host environment rather than by a fixed hierarchy of carbon sources (Chang et al. 2025). Chronic infection adds a temporal dimension because bacterial populations can adapt to resources generated by tissue remodeling.

During chronic infection, *S. aureus* can evolve increased exploitation of collagen-derived proline. Clinical adaptation is associated with enhanced proline acquisition and metabolism, while host tissue remodeling increases access to collagen-derived substrate (Urso et al. 2024). Tissue repair increases access to collagen-derived proline, while adaptation can make this substrate more important for bacterial fitness by improving its acquisition and use. The nutritional conditions shaping chronic infection may differ from those encountered when infection was established: both the substrate landscape and the metabolic capabilities of the bacteria occupying it can change over time.

The timing and anatomical scope of this adaptation remain unclear. The clinical isolates span years of chronic infection, but the time required for this metabolic adaptation in vivo has not been measured (Urso et al. 2024). Whether comparable adaptation occurs at infection sites with different patterns of collagen deposition or tissue repair also remains unknown. Together, the transition to serum described above and this adaptation show that the carbon conditions that shape bacterial survival and regrowth can change over the course of an infection. This temporal variation underpins the proposed two-stage model.

### Host carbon use converts nutrient access into antimicrobial function

Carbon acquired by host cells is not simply removed from the bacterial nutrient pool; it can be converted into antimicrobial chemistry (O’Neill et al. 2016). Myeloid GLUT1-dependent glucose uptake sustains NADPH availability and oxidative antimicrobial activity during MRSA skin infection (Banerjee et al. 2024). Loss of myeloid GLUT1 reduces the NADPH pool and oxidative burst and produces unresolved lesions with higher late bacterial burdens, despite preservation of transcriptional machinery for iNOS- and NOX-dependent antimicrobial pathways (Banerjee et al. 2024). The consequence of host glucose acquisition depends on the biochemical function supported after uptake.

Comparable metabolic reprogramming can support different immune outputs in other host cell types. Glycolytic activation contributes to protective keratinocyte responses during *S. aureus* skin infection (Wickersham et al. 2017), whereas metabolic reprogramming of monocytes can promote pro-inflammatory activity and biofilm clearance (Yamada et al. 2020). Conversely, *S. aureus*-induced neutrophil itaconate production can suppress oxidative burst, demonstrating that increased host metabolic activity is not intrinsically antimicrobial (Tomlinson et al. 2023). Increased host use of a carbon pathway does not have a fixed protective value. Its effect on *S. aureus* depends on the biochemical function supported in the responding host cell.

### Bacterial carbon fate feeds back on host metabolic control

Bacterial metabolism can modify the host environment after carbon has been acquired. *S. aureus*-derived lactate can stimulate mitochondrial reactive oxygen species and neutrophil extracellular trap release, providing a route by which a bacterial fermentation product alters neutrophil antimicrobial behavior (Wise et al. 2025). In biofilm-associated infection, bacterial lactate can instead inhibit HDAC11-dependent control of IL-10 and reprogram myeloid responses toward an immunosuppressive state (Heim et al. 2020). The biological effect of a bacterial carbon product depends on the host cell and infection setting in which it is encountered.

Host metabolites can reciprocally redirect bacterial carbon use. *S. aureus* induces an itaconate-dominated host immunometabolic response, and itaconate suppresses bacterial glycolysis while redirecting carbon toward biofilm-associated polysaccharide production (Tomlinson et al. 2021). Feedback can also occur when bacterial metabolism removes a host metabolite that constrains the host response. In a Δ*hemB* small-colony-variant model, *S. aureus* induced host glycolysis and necroptosis while increasing bacterial *fumC* expression by more than two orders of magnitude (Wong Fok Lung et al. 2020). FumC-dependent depletion of local fumarate was linked to failure of trained immunity and loss of protection against a secondary skin challenge (Wong Fok Lung et al. 2020). Carbon exchange at the interface can modify host-cell metabolism not only through secreted bacterial products such as lactate, but also through bacterial consumption of host immunometabolites (Wise et al. 2025; Wong Fok Lung et al. 2020).

The carbon environment encountered later in infection can reflect earlier host and bacterial metabolism. Host glucose utilization changes antimicrobial activity, bacterial lactate modifies immune-cell behavior, and host itaconate redirects bacterial carbon use toward biofilm-associated metabolism (Banerjee et al. 2024; Heim et al. 2020; Tomlinson et al. 2021). As these processes accumulate, the physiological state of *S. aureus* at a given time need not reflect the extracellular carbon conditions present when infection was established.

Host cells and *S. aureus* are not the only consumers or producers capable of changing local carbon chemistry (Maslova et al. 2024). In polymicrobial wounds, competition for carbon sources can alter community structure and infection progression (Maslova et al. 2024). In airway-associated models, the commensal-derived short-chain fatty acids propionate and butyrate alter *S. aureus* branched-chain-fatty-acid metabolism and lower the branched- to straight-chain fatty-acid ratio (Fletcher et al. 2026). They compromise membrane homeostasis, suppress *agr* signaling and increase susceptibility to membrane-targeting antimicrobials, while reducing competitive fitness against *P. aeruginosa* (Fletcher et al. 2026). Commensals can change this environment by consuming extracellular substrates and by altering the membrane lipid composition that shapes antibiotic susceptibility and interspecies fitness (Fletcher et al. 2026). These measurements do not directly quantify intracellular precursor flux. These findings illustrate how commensals can modify antibiotic susceptibility under the conditions tested. Whether the effect extends across microbiomes or antimicrobial classes remains unknown.

## Carbon availability affects antibiotic killing through the physiological state it produces

Carbon-conditioned physiology can change antibiotic killing in opposite directions. Host-imposed glucose restriction can lower bacterial ATP and reduce killing within macrophages (Beam et al. 2023), whereas provision of a metabolically usable carbon source can increase killing of defined persister populations (Prax et al. 2016; Allison et al. 2011; Xing et al. 2025). Productive use of host-derived lipids can instead support a protective biofilm and envelope phenotype (Ledger et al. 2026). The relationship between carbon availability and treatment efficacy depends on the bacterial function altered and the antibiotic whose action requires or is opposed by that function.

Metabolic rewiring refers here to a dynamic reorganization of pathway activity and carbon routing that changes bacterial energy production, redox balance, biosynthesis or envelope composition without itself constituting heritable resistance (Acierno et al. 2025). We use the term only when pathway-level reorganization is demonstrated and treat reversibility as an experimental property rather than part of the label; where reversibility was not measured, we refer instead to an altered metabolic state. The examples below include changes initiated by host nutrient competition, immune metabolites, respiratory damage and productive use of host lipids. Whereas recent rewiring frameworks connect transient physiological survival conceptually with later resistance, the present analysis separates immediate killing from post-treatment population recovery and makes cellular ancestry an explicit test of a connection that remains to be demonstrated.

### Host-imposed constraints on antibiotic killing

A direct example occurs within macrophages. *S. aureus* α-toxin activates the NLRP3 (NOD-, LRR- and pyrin domain-containing protein 3) inflammasome, a cytosolic innate-immune signaling complex, and increases host glycolytic activity, reducing cytoplasmic glucose available to intracellular bacteria (Beam et al. 2023). Bacteria exposed to this glucose-restricted environment contain less ATP and survive antibiotic treatment more efficiently. Pharmacological inhibition or genetic loss of NLRP3 reduces macrophage glycolysis. Inhibiting NLRP3 or restoring cytoplasmic glucose increases bacterial ATP and improves antibiotic killing (Beam et al. 2023). These interventions link host glucose consumption, bacterial energetic state and treatment efficacy, rather than inferring tolerance from nutrient restriction alone. This causal chain is resolved for intracellular bacteria in a defined macrophage setting; it does not establish the same mechanism in extracellular populations or separate the metabolic effect of NLRP3 perturbation from its broader inflammatory consequences.

Host glucose restriction has competing effects on bacterial survival and antibiotic efficacy. Increased macrophage glycolysis limits a substrate that can support bacterial metabolism, but the accompanying decrease in bacterial ATP also reduces antibiotic killing among surviving intracellular bacteria. In this setting, restricting bacterial access to glucose simultaneously imposes metabolic stress and produces a physiological state less susceptible to treatment.

Reduced bacterial energetic activity can arise without carbon restriction. During systemic infection, macrophage-derived reactive oxygen species damage bacterial iron–sulfur-cluster-containing proteins, including TCA-cycle enzymes, reducing respiration and ATP production and increasing tolerance to multiple antibiotics (Rowe et al. 2020). The initiating constraint lies downstream of substrate availability: host antimicrobial chemistry impairs the bacterial machinery required to convert available nutrients into respiratory energy. Metabolically active, non-dividing intracellular *S. aureus* persisters also survive antibiotics reversibly, showing that persistence cannot be equated with complete metabolic inactivity (Peyrusson et al. 2020). Whether the same ROS–respiration pathway dominates tolerance in other anatomical niches remains unclear.

Host-derived itaconate produces another metabolically attenuated state. Exposure of *S. aureus* to itaconate slows growth, decreases intracellular ATP and alters glycolysis, the TCA cycle, pyruvate metabolism and arginine biosynthesis (Zhao et al. 2024). These changes increase survival during exposure to gentamicin, amikacin and kanamycin without increasing MIC. In infected THP-1 macrophages, *S. aureus* increases expression of *IRG1*/*ACOD1*, which encodes the cis-aconitate decarboxylase responsible for itaconate synthesis, and siRNA-mediated reduction of *IRG1* expression decreases bacterial survival during aminoglycoside treatment (Zhao et al. 2024). The effect is neither permanent nor antibiotic-independent: comparable protection was not observed with ciprofloxacin or vancomycin under the conditions tested, and the survival difference disappeared after 24 h of antibiotic exposure (Zhao et al. 2024).

### Carbon utilization and metabolic sensitization or protection

Energy state can also determine killing by controlling antibiotic entry rather than only the activity of an intracellular target. Aminoglycoside uptake is promoted by an energized membrane: metabolizable substrates generate proton motive force (PMF) that increases drug internalization and killing without requiring growth resumption. In *S. aureus* persisters, fructose enabled PMF-dependent gentamicin killing (Allison et al. 2011), and a recent genome-wide screen showed that glycerol potentiated several aminoglycosides through GlpK-initiated catabolism, increased PMF and enhanced uptake in stationary-phase cells, biofilms, clinical isolates and a murine skin-infection model (Xing et al. 2025). These results provide a mechanistic counterpoint to itaconate-induced aminoglycoside tolerance: carbon use can sensitize a low-activity population when it restores the energetic process required for drug entry. The inference is antibiotic-specific, because the same metabolic input need not improve uptake or killing by drugs whose action is not limited by PMF.

Carbon utilization can also reduce antibiotic susceptibility without producing a low-energy state. Exposure to human serum primes *S. aureus* for biofilm formation and increased tolerance to vancomycin and daptomycin through utilization of host triglycerides (Ledger et al. 2026). The Geh lipase releases glycerol from serum lipids, which promotes increased synthesis of D-alanylated wall teichoic acids and biofilm development; inhibition of either Geh activity or wall-teichoic-acid synthesis reduces biofilm formation and restores antibiotic susceptibility (Ledger et al. 2026). The protective phenotype arises from productive use of a host-derived carbon resource for cell-envelope and biofilm remodeling, without evidence of carbon deprivation or energetic collapse. The mechanism was defined after serum priming and in biofilm-associated assays; its quantitative contribution during infection and its effect on planktonic populations remain unresolved.

Carbon restriction can reduce antibiotic killing when it lowers bacterial energetic activity, but low-energy physiology can also arise when host-derived stress disrupts central metabolism despite continued substrate access. Carbon acquisition can produce the opposite metabolic condition yet still impair treatment when the acquired substrate supports protective cell-envelope or biofilm remodeling. The same change in extracellular carbon availability cannot be expected to alter all antibiotics in the same direction, because treatment efficacy depends on the bacterial functions sustained or suppressed under that metabolic condition.

These mechanisms should not be treated as mutually exclusive. Carbon restriction, impaired respiration, redox imbalance, slowed activity of antibiotic-relevant processes and protective envelope remodeling can coexist within a population and contribute differently across cells and antibiotics. The unit of inference is a physiological state within a specified niche–antibiotic combination.

## Carbon availability reshapes the fitness landscape of antimicrobial resistance

Because tolerance and resistance describe different responses to antibiotic exposure, carbon conditions can affect them through several population-level processes. These include the number of cells available to generate mutations, survival through an antibiotic bottleneck, selection of pre-existing variants, fitness costs of resistant variants and post-treatment expansion. A metabolic environment can change the probability that a resistant lineage becomes detectable without changing the per-cell rate at which resistance-conferring mutations arise. Because resistance mutations frequently impose fitness costs that vary with the environment, the relative success of resistant and susceptible genotypes can also change when the environment changes even if their resistance determinants remain unchanged (Andersson and Hughes 2010).

### Hyperglycaemia and expansion under antibiotic selection

This distinction is experimentally accessible in diabetic skin infection. During rifampicin treatment of *S. aureus* skin and soft-tissue infection, rifampicin-resistant populations emerge rapidly in hyperglycaemic diabetic mice but remain undetectable over the corresponding treatment period in normoglycaemic animals (Shook et al. 2025). Resistant isolates recovered from diabetic infections retain high rifampicin MICs after isolation, establishing a heritable resistance phenotype rather than transient antibiotic tolerance. Population sequencing did not reveal genomic signatures of increased mutation rates in diabetic infection (Shook et al. 2025).

The difference instead develops at the population level. Hyperglycaemic infection supports greater bacterial burdens and rifampicin-resistant population expansion during treatment (Shook et al. 2025). Rifampicin was started 24 h after inoculation, whereas the principal burden measurements were made on day 5; these measurements cannot determine the burden immediately before treatment. Lowering blood glucose with insulin reduces bacterial burden and markedly limits recovery of rifampicin-resistant bacteria. The rifampicin-resistant isolate D6R shows no competitive disadvantage against JE2 in vitro or in macrophages, and competition does not differ between diabetic and normoglycaemic infection environments (Shook et al. 2025). A separate clinical vancomycin-intermediate *S. aureus* isolate, VISA1984, is growth-attenuated in normoglycaemic infection but proliferates more extensively and forms larger lesions in diabetic hosts (Shook et al. 2025). Thus, the diabetic environment supports rifampicin-resistant population expansion and can separately improve the performance of an attenuated resistant genotype. These interventions nevertheless occur in a streptozotocin-induced diabetic host, in which hyperglycaemia, endocrine correction and immune physiology change together; they do not isolate extracellular glucose as the sole causal variable.

Elevated glucose also alters the outcome of rifampicin exposure at the airway epithelium, although the evolutionary process is less completely resolved. Increasing basolateral glucose from 5.5 to 12.5 mM does not alter bacterial burden during the first 6 h of infection in air–liquid interface cultures of non-CF 16HBE and CFBE41o− bronchial epithelial cells (Hughes et al. 2025). However, it increases the formation of large *S. aureus* aggregates specifically on CF epithelial cells. After rifampicin treatment, hyperglycaemia abolishes the reduction in total bacterial burden otherwise observed in CF cultures and increases recovery of colonies capable of growing on rifampicin-containing agar in both CF and non-CF epithelial models. Restricting airway-surface glucose with 2-deoxyglucose reduces both aggregation and recovery of these colonies (Hughes et al. 2025).

The plating assay provides lower-resolution evolutionary evidence than the diabetic skin-infection study. It demonstrates that hyperglycaemia increases recovery of bacteria able to grow under rifampicin selection, but the recovered colonies were not characterized for persistence of elevated MIC after removal of rifampicin. Altered mutation supply, selection of pre-existing variants and differences in relative fitness during treatment remain uncertain. Elevated glucose alone also fails to reproduce the epithelial phenotype in conventional broth culture, placing the effect within the host-associated environment rather than supporting a direct glucose-to-resistance mechanism (Hughes et al. 2025).

### Metabolic phenotypes of resistant bacteria

Resistance can also remodel carbon metabolism after the resistant phenotype has evolved. Laboratory selection of *S. aureus* Newman derivatives for ciprofloxacin, cefazolin or dual resistance produces strains with broad suppression of central carbon and energy metabolism, including reduced activities of pyruvate dehydrogenase and several TCA-cycle enzymes together with lower NADH and ATP (Pan et al. 2026). Supplementation with pyruvate, citrate or fumarate can enhance ciprofloxacin or cefazolin killing of the corresponding resistant populations, although the magnitude and concentration dependence differ among metabolites and antibiotics.

These experiments characterize a metabolic phenotype of laboratory-evolved resistant bacteria and demonstrate that manipulating central metabolism can modify their subsequent antibiotic susceptibility. Because metabolic measurements were performed after serial selection under antibiotic pressure, the experiments resolve neither carbon restriction as the origin of the resistant genotype nor the direction of causality between the selected mutations and metabolic suppression. Reduced central carbon metabolism may be a consequence or stabilizing feature of an evolved resistant state rather than evidence that a carbon-poor environment-initiated resistance.

A complementary genetic perturbation demonstrates that central carbon metabolism can simultaneously organize energy state, virulence and antimicrobial susceptibility. In MRSA BAA1717, deletion and complementation analyses identified aconitase AcnA as a functional nexus (Li et al. 2025). Δ*acnA* abolished staphyloxanthin production and haemolytic activity, disrupted cell-wall structure and increased autolysis. ATP, NADPH and membrane potential decreased, while MICs to β-lactams, aminoglycosides, macrolides and vancomycin fell by 2- to 128-fold (Li et al. 2025). Virulence-associated transcripts (including *crtO*; *crtP*; *hla*; *hlgA*; and *hlyIII*) and resistance-associated transcripts (including *sepA*; *norA*; *ftsW*; *pbp* genes; *murC*; and *lytR*) were also reduced. These results provide direct genetic evidence that a TCA-cycle enzyme can coordinate carbon-derived energetic and redox functions with virulence and multidrug susceptibility. The study identifies a metabolic control point, but its genetic design does not determine whether extracellular carbon availability regulates AcnA in vivo or whether physiological carbon restriction phenocopies *acnA* deletion. Because the evidence derives from MRSA BAA1717 and deletion of a pleiotropic TCA-cycle enzyme, conservation across lineages and attribution of every downstream phenotype specifically to carbon flux require independent testing.

Carbon availability can alter the population dynamics through which heritable resistance becomes established without a demonstrated direct mutagenic effect. In diabetic skin infection, hyperglycaemia favors rifampicin-resistant population expansion under antibiotic selection without genomic evidence of hypermutability (Shook et al. 2025). The improved performance of VISA1984 in diabetic hosts is a separate example of a host-dependent resistant phenotype. In airway infection, elevated glucose also increases recovery of bacteria capable of growth under rifampicin selection, but the evolutionary process producing that population has not been determined. Once resistance has evolved, central carbon metabolism can itself be remodeled, making metabolic state both a potential modifier of selection and a phenotype of the selected population. The *acnA* perturbation further shows that carbon-metabolic architecture can directly coordinate virulence and baseline multidrug susceptibility, while its genetic design prevents inference that extracellular carbon conditions initiated either phenotype.

## Treatment failure may emerge from transitions between restrictive and permissive metabolic states

The two-stage model separates survival during treatment from the fate of survivors after a change in metabolically usable carbon (Fig. 1b). Stage 1 captures antibiotic exposure under a carbon-conditioned physiological state that changes bacterial killing. Stage 2 describes the later interval in which carbon restoration or rerouting can restore killing, permit susceptible regrowth or alter expansion of resistant variants. These are analytical stages, not an obligatory biological sequence, and the proposed descent of Stage 2 populations from Stage 1 survivors is an explicit hypothesis rather than an assumption of the model. The distinction is needed because carbon availability can change as host metabolism, tissue damage, inflammation and bacterial adaptation alter both the substrates accessible to *S. aureus* and the capacity of the bacterium to use them. A population surviving one treatment interval may encounter a different nutritional environment before subsequent recovery or selection.

### Survival and recovery across nutritional transitions

Nutrient restriction can generate a long-lived starvation-survival state independently of antibiotic exposure. Glucose limitation causes a rapid loss of viability in *S. aureus*, but a small surviving population can remain culturable for months (Watson et al. 1998). Cells of strain 8325-4 maintained for seven days under glucose limitation resume growth after nutritional upshift with complex medium or with glucose and amino acids, whereas either nutrient class alone is insufficient. RNA synthesis resumes immediately, protein synthesis within minutes and chromosome replication before the first cell division, demonstrating an ordered metabolic recovery rather than simple outgrowth of continuously proliferating cells (Clements and Foster 1998). Nutritional restriction can leave a surviving population capable of returning to active growth when an appropriate combination of carbon and other nutrients becomes available.

Antibiotic susceptibility can change during this recovery. Addition of glucose to stationary-phase *S. aureus* persisters increases daptomycin killing within one hour, showing that carbon availability can modify susceptibility before substantial population expansion is required (Prax et al. 2016). As discussed above, glucose consumption by host cells lowers ATP levels in intracellular bacteria and increases their survival during antibiotic exposure (Beam et al. 2023). Together, these observations show that metabolic reactivation need not benefit the surviving population. Renewed carbon use can restore physiological processes required for antibiotic killing. Nutritional recovery and bacterial regrowth are not equivalent outcomes.

### Tolerance, recovery and resistance evolution

Tolerance has no fixed evolutionary consequence. In clinical *S. aureus* populations, tolerance preceded resistance during combination treatment, demonstrating that this evolutionary connection is already established beyond single-drug laboratory selection (Liu et al. 2020). GdpP is the phosphodiesterase that degrades cyclic di-AMP (c-di-AMP), a bacterial second messenger whose accumulation in *S. aureus* alters cell size and cell-envelope stress physiology (Corrigan et al. 2011). Loss of GdpP function provides a genetically defined way to alter c-di-AMP signaling before antibiotic selection is considered. In several *S. aureus* backgrounds, *gdpP* loss-of-function increases intracellular c-di-AMP and produces β-lactam tolerance without increasing β-lactam MICs (Poon et al. 2022). During serial β-lactam exposure, *gdpP* mutations can arise before mutations associated with high-level resistance, and an isogenic Δ*gdpP* strain evolves resistance to nafcillin and cefoxitin more rapidly than its susceptible parental strain (Poon et al. 2022). A tolerance phenotype can increase the opportunity for subsequent resistance evolution under at least one defined signaling mechanism, but the experiment does not establish carbon restriction as the cause of that evolutionary sequence.

Precedents beyond *S. aureus* show that carbon transitions can generate persisters and that persistence can alter evolutionary opportunity. In *Escherichia coli*, glucose exhaustion during a carbon-source transition activated a ppGpp-linked pathway that increased fluoroquinolone persister formation (Amato et al. 2013). Under intermittent ampicillin exposure, tolerance mutations preceded resistance and reduced extinction of lineages carrying emerging resistance mutations (Levin-Reisman et al. 2017). Recovery after ofloxacin exposure can also increase resistance development through RecA, the SOS response and DNA polymerase V (UmuDC), identifying a drug-specific mutagenic route in persister descendants (Barrett et al. 2019). Across laboratory and natural *E. coli* isolates, higher persistence was associated with a greater probability of resistance evolution through both increased survival and higher mutation rates (Windels et al. 2019). These mechanisms should not be assumed to operate together across species or metabolic settings. Population sequencing found no genomic signatures of increased mutation rates in diabetic infection with *S. aureus* (Shook et al. 2025). In the latter model, the increased opportunity for resistance is instead supported by population expansion and altered selection. The lineage hypothesis asks a further causal question: whether cells surviving an earlier carbon-conditioned state contribute disproportionately to the population recovered later, irrespective of whether resistance arises through increased per-cell mutagenesis or selection within an expanded population.

The carbon environment can also constrain the resistance trajectory itself. In *E. coli* evolved under three antibiotics, growth on glucose or acetate altered the speed and mode of resistance acquisition and the compensatory metabolic states of resistant populations (Zampieri et al. 2017). That result demonstrates that carbon source can shape evolutionary paths, but it does not identify a carbon-restricted survivor as the ancestor of a later resistant population. The lineage-specific hypothesis proposed here asks whether carbon-conditioned survivors in *S. aureus* infection contribute disproportionately to the population recovered after metabolic reactivation and continued antibiotic selection.

The same relationship is not reproduced by all tolerant states. Repeated daptomycin exposure and regrowth generate both tolerant and resistant derivatives of methicillin-resistant *S. aureus* (Sulaiman and Lam 2021). The highly tolerant TOL6 derivative carries a substantial fitness cost and does not show the increase in daptomycin MIC observed in the parental population during subsequent cycles. The parental strain develops a two- to fourfold increase in MIC over these cycles, whereas TOL6 does not show a corresponding increase. Survival during antibiotic exposure is insufficient to predict evolutionary outcome: the fitness of surviving bacteria during subsequent growth can constrain their contribution to the population available for further selection.

Selection after recovery provides another boundary. In *E. coli*, three persister cycles with recovery in antibiotic-free medium maintained the baseline ampicillin MIC of 8 µg/mL. Adding sub-MIC ampicillin after recovery increased MIC to 16 µg/mL in the third cycle, without detectable genetic fixation after quality filtering (Park et al. 2026). Thus, repeated persistence alone was insufficient to change susceptibility under these conditions, while additional selection altered the phenotypic outcome. The MIC increase does not by itself demonstrate fixation of resistance mutations in persister descendants.

The prior frameworks explain different parts of the proposed transition. Tolerance can precede resistance during intermittent treatment and in clinical *S. aureus* infection under drug combinations (Levin-Reisman et al. 2017; Liu et al. 2020). After fluoroquinolone exposure, *E. coli* persister descendants can develop resistance through an SOS-associated DNA damage response (Barrett et al. 2019), whereas intracellular *S. aureus* persisters demonstrate reversible survival and recovery without establishing subsequent resistance (Peyrusson et al. 2020). These precedents connect survival with later population outcomes, but do not identify host-regulated carbon access or bacterial substrate fate as the determinant of which survivors seed those outcomes. Our two-stage model makes that additional link testable across a metabolic transition. It leaves open whether the recovered lineage remains susceptible, acquires resistance or is outcompeted under subsequent selection. Carbon availability may influence these outcomes by changing how many cells recover and the relative success of susceptible and resistant variants during regrowth.

In diabetic skin infection, hyperglycaemia increases *S. aureus* population size and recovery of rifampicin-resistant bacteria without genomic evidence of hypermutability (Shook et al. 2025). A metabolically permissive environment can increase the population exposed to antibiotic selection and alter the relative success of resistant variants once they are present. It remains unknown whether resistant cells expanding in hyperglycaemic tissue descend from bacteria that previously survived a carbon-restricted, antibiotic-tolerant state.

### Carbon-conditioned survival and the lineage hypothesis

Taken together, these experiments place carbon conditions at different causal levels. Host glucose competition, ROS-mediated TCA-cycle damage and itaconate modify immediate antibiotic killing by changing bacterial physiology. Serum-triglyceride utilization produces a protective state through cell-envelope remodeling, whereas hyperglycaemia favors rifampicin-resistant expansion and improves the performance of a separate attenuated VISA isolate. None of these findings alone connects extracellular carbon availability, a tolerant survivor and a later resistant descendant. Reading the evidence across systems requires distinguishing the mechanism demonstrated in each experiment from the broader evolutionary sequence it may suggest (Table 1).

**Table 1:** Experimental systems linking metabolic context to *Staphylococcus aureus* physiology and antibiotic response

Study and experimental model	Metabolic context or perturbation	Evidence category	Demonstrated outcome	Mechanistic link resolved	Inferential boundary
Garnett et al. 2014 – Polarized airway epithelial model	Basolateral sugar supply and epithelial sugar transport	No antibiotic outcome assessed	Epithelial transport limits sugar accumulation in airway-surface liquid and thereby constrains sugar-supported bacterial growth	Host regulation of the extracellular substrate pool available at the epithelial surface	Does not quantify host-versus-bacterial carbon partitioning or the intracellular fate of acquired bacterial carbon
Thurlow et al. 2020 – Hyperglycaemic murine skin infection	Diabetes-associated hyperglycaemia and altered host and bacterial glucose use	No antibiotic outcome assessed	Diabetic abscesses contain more glucose; impaired phagocyte glucose uptake reduces respiratory-burst activity, whereas bacterial glucose import supports virulence and infection severity	Competition for glucose links host antimicrobial metabolism to bacterial fitness	The diabetic phenotype combines altered immunity and nutrient availability and cannot be reduced to extracellular glucose concentration alone
Mujchariyakul et al. 2026 – Five clinical lineages exposed to human serum, with JE2 mutant validation	Transition from laboratory medium to acellular human serum	No antibiotic outcome assessed	Serum elicits coordinated responses in carbon use, TCA-cycle activity, iron acquisition and oxidative-stress defense; *gapdhB* and *sucA* contribute to serum fitness	Carbon metabolism is integrated with iron acquisition and stress adaptation in serum	Acellular serum does not reproduce whole-blood immunity or track dissemination from an infection focus into the bloodstream
Ledger et al. 2022 – Human-serum adaptation	LL-37–GraRS signaling; increased membrane cardiolipin	Physiological tolerance	Serum adaptation increases daptomycin tolerance through peptidoglycan accumulation and a separate cardiolipin-dependent response	Host signals and membrane remodeling alter killing	Does not establish a carbon cause for the full serum response
Urso et al. 2024 – Sequential clinical isolates and chronic airway-infection models	Collagen-derived proline released in damaged airway tissue	No antibiotic outcome assessed	Late chronic isolates exhibit enhanced proline acquisition and catabolism, supporting growth on collagen-rich host substrates	Within-host adaptation changes the fitness value of a tissue-derived carbon source	Does not establish how rapidly this adaptation arises or whether it generalizes beyond the airway context studied
Fletcher et al. 2026 – Airway-associated in vitro and polymicrobial models	Commensal-derived propionate and butyrate	Antibiotic susceptibility	SCFAs remodel branched-chain fatty-acid metabolism, impair membrane homeostasis and *agr* signaling, sensitize *S. aureus* to membrane-targeting antimicrobials and reduce competitive fitness	SCFAs alter membrane lipid composition and antimicrobial susceptibility	Does not measure precursor flux or establish heritable resistance; metabolite and antimicrobial specificity limit extrapolation
Beam et al. 2023 – Intracellular macrophage and infection models	NLRP3-driven host glycolysis and depletion of macrophage cytoplasmic glucose	Physiological tolerance	Intracellular bacterial ATP decreases and antibiotic survival increases; restoring glucose availability or inhibiting NLRP3 improves killing	Host-imposed carbon restriction links bacterial energetic state to antibiotic killing	Does not establish a universal carbon-restriction mechanism for tolerance across antibiotics, cell types or infection niches
Rowe et al. 2020 – Macrophage and murine systemic-infection models	Host ROS-mediated damage to iron–sulfur proteins and TCA-cycle functions	Physiological tolerance	Respiration and ATP decrease, accompanied by slower killing by multiple antibiotics in macrophages and during systemic infection	A low-energy tolerant state can arise from host-induced metabolic damage without extracellular carbon depletion	Does not implicate extracellular carbon restriction as the initiating cause
Peyrusson et al. 2020 – Intracellular *S. aureus*	Antibiotic exposure in infected macrophages	Persistence	Biphasic killing leaves non-dividing, metabolically active persisters; susceptibility and growth recover after antibiotic removal	Reversible intracellular persistence without complete metabolic inactivity	Does not trace a defined carbon source or demonstrate subsequent resistance
Zhao et al. 2024 – In vitro cultures and THP-1 macrophage model	Host-derived itaconate	Physiological tolerance	Growth and ATP decrease and aminoglycoside survival increases without an MIC increase; the effect disappears by 24 h	An immunometabolite induces a reversible physiological state that reduces aminoglycoside killing	The effect is antibiotic- and time-dependent and does not establish a general tolerance phenotype
Xing et al. 2025 – Stationary-phase cells, biofilms, clinical isolates and murine skin infection	Glycerol co-treatment with aminoglycosides and GlpK-dependent catabolism	Persistence	Glycerol rapidly increases PMF-dependent aminoglycoside uptake and killing	GlpK-initiated use of metabolically available glycerol links carbon catabolism to PMF, drug uptake and killing	The high glycerol concentration and brief co-treatment do not establish endogenous host glycerol availability or generalization beyond aminoglycosides
Ledger et al. 2026 – Human-serum exposure	Geh-dependent triglyceride hydrolysis and utilization of lipid-derived glycerol	Physiological tolerance	Serum exposure promotes D-alanylated wall teichoic acids, biofilm formation and reduced vancomycin and daptomycin killing	Productive use of host-derived carbon supports protective cell-envelope remodeling	Does not support a low-energy or carbon-deprivation mechanism and does not establish the in vivo contribution of this pathway
Shook et al. 2025 – Diabetic murine skin infection	Diabetes-associated hyperglycaemia; rifampicin treatment from 24 h; separate VISA1984 infections	Heritable resistance	Rifampicin resistance expands without genomic evidence of hypermutability. D6R has no demonstrated competitive cost; VISA1984 grows better in diabetic than normal hosts.	Population opportunity for rifampicin resistance; separate host-context effect on an attenuated VISA isolate	Does not show compensation of a D6R fitness cost, greater burden before treatment, or descent from carbon-restricted survivors
Hughes et al. 2025 – CF and non-CF airway epithelial air–liquid-interface models	Basolateral glucose at 5.5 versus 12.5 mM	Recovery under selection	Hyperglycaemia increases aggregation and recovery on rifampicin-containing agar; limiting glucose reverses these effects	Host-associated glucose availability modifies bacterial recovery after antibiotic exposure	Does not establish stable resistance after isolation, distinguish mutation supply from selection, or demonstrate the same mechanism as diabetic skin infection
Pan et al. 2026 – Laboratory-evolved, antibiotic-resistant Newman derivatives	Resistance-associated metabolic remodeling and supplementation with pyruvate, citrate or fumarate	Heritable resistance; Antibiotic susceptibility	Resistant derivatives show suppressed central carbon and energy metabolism; selected metabolites increase antibiotic killing in defined strain–drug combinations	Resistance-associated metabolism can create exploitable energetic vulnerabilities	Does not show that carbon restriction caused the acquisition of resistance or that metabolite supplementation will generalize across strains and antibiotics
Li et al. 2025 – MRSA BAA1717 Δ*acnA* mutant and genetic complementation	Genetic disruption of the TCA-cycle aconitase AcnA	Antibiotic susceptibility	Loss of *acnA* abolishes staphyloxanthin and haemolytic activity, lowers ATP, NADPH and membrane potential, disrupts the cell wall and decreases MICs by 2- to 128-fold	Genetic link between central metabolism, virulence and multidrug susceptibility	Genetic deletion does not demonstrate regulation by extracellular carbon availability or a carbon-driven evolutionary route to resistance
Prax et al. 2016 – Stationary-phase persisters in vitro	Glucose supplementation during daptomycin exposure	Persistence	Glucose metabolism accelerates daptomycin killing of stationary-phase persisters	Metabolic reactivation can restore killing by a defined antibiotic	The effect is not explained by proton-motive force and does not generalize to all antibiotic classes or infection settings
Liu et al. 2020 – Clinical *S. aureus* populations and evolution experiments	Combination treatment and evolving tolerance	Physiological tolerance; Heritable resistance	Tolerance emerges before resistance; subsequent resistance undermines protection expected from drug combinations	Clinical and experimental precedent for tolerance facilitating resistance evolution	Does not manipulate carbon allocation or connect a defined carbon-restricted founder with resistant descendants
Poon et al. 2022 – Defined *gdpP* mutants and serial β-lactam exposure	Loss of GdpP function and increased c-di-AMP signaling rather than carbon restriction	Physiological tolerance; Heritable resistance	Loss of *gdpP* produces β-lactam tolerance without an MIC increase and facilitates subsequent resistance during serial exposure	A defined tolerant state can increase later evolutionary opportunity	Does not establish carbon restriction as the origin of either tolerance or resistance and does not make tolerance a universal precursor of resistance
Sulaiman and Lam 2021 – Adaptive laboratory evolution under repeated daptomycin exposure	Repeated cycles of antibiotic killing and regrowth	Physiological tolerance; Heritable resistance	Evolved phenotypes differ in survival and fitness, and the most tolerant derivative does not acquire the MIC increase observed in the parental population	Survival advantage alone does not determine which lineage ultimately expands	Does not establish a fixed tolerance-to-resistance sequence or a carbon-dependent mechanism

Studies are ordered by their first substantive discussion in the text. Evidence category describes the antibiotic outcome examined, independently of the metabolic mechanism. Physiological tolerance means reduced killing without increased MIC; persistence means survival of a subpopulation supported by the experimental design; heritable resistance means stable resistance after isolation with supporting susceptibility evidence. Recovery under selection means growth on antibiotic-containing medium without confirmed heritability. Antibiotic susceptibility means a drug-response change not established as one of those phenotypes; no antibiotic outcome assessed identifies studies without an antibiotic endpoint. Categories describe the investigated outcome, including its attenuation or reversal, rather than a uniform direction of effect. A study addressing distinct outcomes can receive more than one category

Abbreviations: ALI, air–liquid interface; BCFA, branched-chain fatty acid; CF, cystic fibrosis; MIC, minimum inhibitory concentration; MRSA, methicillin-resistant *S. aureus*; ROS, reactive oxygen species; SCFA, short-chain fatty acid; TCA, tricarboxylic acid; WTA, wall teichoic acid

Restrictive and permissive carbon states may influence different stages of treatment failure without constituting a fixed sequence. In the proposed two-stage model, a restrictive condition can increase survival during antibiotic exposure when it produces a physiological state poorly susceptible to killing. Subsequent restoration or rerouting of metabolically usable carbon can restore antibiotic killing, permit regrowth without altered susceptibility, or favor resistant-population expansion under selection. These Stage 2 outcomes can coexist among lineages within a heterogeneous population and do not represent an obligatory progression. Tolerance is neither a necessary precursor nor a sufficient predictor of resistance (Fig. 1b).

A stronger, lineage-specific version of this model predicts that cells surviving antibiotic exposure during carbon restriction contribute disproportionately to populations recovered after metabolically usable carbon is restored and, under continued antibiotic selection, to the resistant fraction of those populations. This prediction remains untested in *S. aureus* infection. Longitudinal detection of tolerance followed by resistance would not establish it, because temporal succession does not demonstrate cellular descent. Testing the prediction requires bacteria surviving a carbon-restricted, antibiotic-tolerant state to be distinguished from contemporaneous populations, followed through metabolic recovery, and linked to their descendants during subsequent antibiotic selection.

### Falsifiable predictions of the lineage model

The lineage-specific prediction would be rejected if:

1. metabolic reactivation eliminated the earlier tolerant population before substantial regrowth;
2. descendants of tolerant survivors contributed no more than expected from their initial abundance to the recovered population; or.
3. resistant variants arose predominantly from lineages that had not occupied the earlier tolerant state.

Discriminating among these outcomes requires experiments that preserve substrate identity, cellular identity, intracellular carbon fate and ancestry across metabolic recovery.

## An experimental roadmap for linking carbon allocation to antibiotic response across time

Single-cell tracking already follows bacterial survival and recovery through changing conditions; the missing link is with substrate origin and intracellular carbon fate. Extracellular carbon measurements cannot test the lineage-specific prediction because the same concentration can result from different rates of substrate delivery, host uptake, bacterial acquisition and metabolic conversion. The experimental problem is to preserve four pieces of information that are usually separated: substrate identity, the acquiring cell, intracellular carbon fate and ancestry through recovery. Existing approaches resolve different parts of this problem; their value depends on the biological transition they can document, not analytical sensitivity alone.

### Following cells through nutrient changes and antibiotic exposure

Time-lapse microscopy and microfluidics provide direct continuity between survival and outgrowth. Individual *E. coli* persisters can be followed through antibiotic exposure and recovery, and lineage-retaining platforms accommodate nutrient changes and entry into or exit from stationary phase (Balaban et al. 2004; Bakshi et al. 2021). Extended histories also reveal persisters that were growing before treatment, so prior growth arrest cannot serve as a universal identifying criterion (Umetani et al. 2025). In *S. aureus*, bio-sCAPA follows individual cells through nutrient stress, antibiotic exposure and regrowth; tolerant survivors show prolonged lag rather than marked differences in subsequent growth rate (Boggon et al. 2023). These observations supply a direct foundation for the proposed transition, but do not identify the carbon source supporting each cell.

Indexed single-cell sorting offers a complementary route when continuous imaging is impractical. Fluorescent phenotypic profiling and backtracing link an individual *S. aureus* cell to subsequent outgrowth, with recovery depending on medium and observation time (Hira et al. 2024). Intracellular *S. aureus* models additionally connect dormancy depth and host oxidative stress with subsequent lag, providing a host-associated precedent (Peyrusson et al. 2022). These methods can identify founders and recovery phenotypes. Combining them with the measurements below requires distinguishing repeat observations in the same living cell from destructive analyses in matched populations.

### Substrate fate and physiological state

Identifying the fate of a defined substrate requires a tracer that remains distinguishable after uptake. In *S. aureus* USA300 LAC, ¹³C₄-fumarate tracing detects fumarate-derived carbon in malate and downstream TCA-cycle intermediates and subsequently in gluconeogenic intermediates, UDP-N-acetylglucosamine, ribose-5-phosphate, nucleotides and NAD(H) (Chen et al. 2025). Loss of FumC changes this labeling pattern and prevents normal entry of fumarate-derived carbon into several downstream pathways, demonstrating substrate routing rather than merely an association between extracellular fumarate and phenotype (Chen et al. 2025). The experiment was performed in bacterial culture, so it shows what *S. aureus* can do with acquired fumarate without determining how a labeled host-accessible pool is partitioned between host and bacterial cells during infection.

Cellular attribution becomes essential when host and pathogen can acquire or transform the same local resource. Heavy-water labeling combined with fluorescence in situ hybridization and nanoscale secondary ion mass spectrometry (FISH-NanoSIMS) has resolved individual *S. aureus* cells directly in sputum from people with cystic fibrosis and quantified marked cell-to-cell differences in anabolic activity (Kopf et al. 2016). Species-specific FISH assigns the isotope signal to bacterial cells, while deuterium incorporation from D₂O reports biomass synthesis at single-cell resolution. This solves a different problem from ¹³C substrate tracing: D₂O reports anabolic activity regardless of whether the carbon supporting that activity came from glucose, lactate, amino acids, fatty acids or another substrate. Cellular identity and activity can be resolved without identifying the carbon source that sustained them.

The physiological state associated with carbon use introduces a further layer because cells with different treatment-relevant programs can coexist in the same population. BaSSSh-seq (bacterial single-cell RNA sequencing with split-pool barcoding, second-strand synthesis and subtractive hybridization) captures transcriptional heterogeneity within *S. aureus* biofilms and resolves subpopulations with distinct metabolic, stress-response and virulence programs after exposure to macrophages, neutrophils and granulocytic myeloid-derived suppressor cells (Korshoj and Kielian 2024). The method identifies cellular programs compatible with substrate acquisition or processing but does not measure carbon flux: transporter or enzyme transcripts cannot establish that a particular carbon source entered the cell or quantify its contribution to downstream metabolites. Computational trajectories inferred from transcriptional similarity likewise do not establish that cells assigned to an earlier state are the physical ancestors of cells assigned to a later state.

Living-cell measurements can add temporal information to physiological state without solving substrate origin. Label-free optical metabolic imaging based on endogenous NAD(P)H and FAD fluorescence lifetimes detects rapid metabolic responses in living *S. aureus* and spatially heterogeneous metabolic regions within intact biofilms (Sorrells et al. 2026). Repeated imaging can follow changes in redox-associated physiology over minutes in the same field, avoiding the purely destructive snapshot produced by many metabolomic and isotope-imaging workflows. The optical signal nevertheless reports redox state rather than the molecular source or intracellular route of carbon, and repeated observation of a field is not equivalent to division-resolved lineage tracking.

### Linking survivor identity to subsequent populations

These boundaries become decisive when the biological question changes from coexistence to descent. Demonstrating that carbon-restricted survivors seed a population recovered after metabolic reactivation requires distinguishing them from contemporaneous cells that did not occupy that state. Their recovery must then be followed as metabolically usable carbon becomes available, with descendants remaining identifiable during growth and antibiotic selection. Neither a later resistant isolate nor a longitudinal sequence of bulk measurements can reconstruct that ancestry. A tolerant population followed in time by a resistant population is compatible with, but does not demonstrate, a tolerance-to-resistance lineage transition.

Chromosomal barcoding provides a partial technical precedent. Heritable barcodes introduced into approximately 450,000 *E. coli* lineages quantify lineage-diversity dynamics during growth under subinhibitory antibiotic pressure (Jasinska et al. 2020). Because barcode inheritance links population changes to clonal ancestry, an adapted strategy could test whether labeled *S. aureus* survivors disproportionately seed post-recovery populations. Barcoding alone would not show that a labeled cell had occupied a carbon-restricted tolerant state; it would need to be coupled to state-specific labeling or isolation, carbon tracing and synchronized antibiotic-killing measurements.

State-associated recording can extend this approach. In *Salmonella*, pSCRATCH uses CRISPR spacer acquisition to mark persisters with a record inherited by their progeny (Rycroft et al. 2024). This supplies a precedent for linking persistence to descendants, although adaptation to *S. aureus* would require validation of marking specificity and fitness under each carbon condition.

A first-generation lineage test can be staged without requiring every variable to be measured simultaneously. A barcoded *S. aureus* population could be exposed to antibiotic under restrictive and matched permissive carbon conditions in a defined host-cell model. Survivors and their barcode frequencies would be enumerated before transfer to a carbon-restoration condition with or without continued antibiotic selection. Barcode enrichment during recovery, antibiotic-killing kinetics and MIC after drug-free regrowth would determine whether Stage 1 survivors contribute more than expected from their initial abundance to the recovered and resistant fractions. Single-cell imaging would follow survival, lag and regrowth, while parallel isotope tracing and ATP or redox measurements in matched aliquots would verify the imposed metabolic transition. These parallel measurements would characterize the corresponding population but could not establish the carbon fate of each imaged founder. This design would not provide simultaneous single-cell measurement of every variable, but it would directly test ancestry while preventing reversible tolerance from being inferred from survival alone and heritable resistance from being inferred solely from growth on drug-containing agar. More complete implementations could subsequently couple state-triggered heritable recording to cell-resolved isotope or optical measurements. The critical advance is the preservation of substrate condition, physiological state and ancestry across the same experimentally controlled transition.

## Conclusion

Carbon availability during *S. aureus* infection cannot be inferred from extracellular substrate concentration alone. Host transport and consumption determine cellular access, bacterial transport determines acquisition, and intracellular metabolic capacity determines the fate of acquired carbon. These processes can vary independently across host compartments and over time. Similar extracellular carbon pools can support different bacterial physiological states, whereas different carbon environments can converge on physiological properties with similar consequences for treatment.

Antibiotic susceptibility is one such consequence, but the relationship is conditional rather than directional. Carbon restriction can accompany physiological states with reduced antibiotic killing when respiration, ATP production or growth are constrained, but tolerance can also arise without carbon depletion, and metabolically productive host-derived substrates can support treatment-refractory states through other mechanisms. Conversely, metabolically usable substrates can increase killing when they restore processes required by a particular antibiotic. Carbon availability modifies antibiotic response through the physiological state it produces; nutrient abundance alone does not predict susceptibility.

The population consequences of changing carbon conditions differ from their immediate effects on killing. Conditions that permit population expansion can increase the opportunity for resistant variants to be recovered. The host environment can independently improve the performance of an attenuated resistant isolate, as shown for VISA1984 in diabetic mice (Shook et al. 2025). Survival during an earlier restrictive state does not show that the surviving cells seed a later resistant population. The lineage-specific prediction that carbon-restricted tolerant survivors disproportionately contribute to populations recovered after carbon restoration remains testable, but it is not a demonstrated evolutionary sequence.

This distinction also constrains therapeutic interpretation. Blocking a bacterial route of host-carbon utilization can be beneficial when that route supports a protective structure. For Geh-dependent use of serum triglycerides, inhibition of Geh or wall-teichoic-acid synthesis reduces biofilm formation and restores vancomycin and daptomycin susceptibility (Ledger et al. 2026). The opposite intervention can be effective when antibiotic action benefits from metabolic reactivation. Glucose accelerates daptomycin killing of stationary-phase *S. aureus* persisters (Prax et al. 2016). Nutrient-based strategies also indicate that restoring defined energetic functions or drug-uptake pathways can recover antibiotic activity in selected experimental settings (Peng et al. 2025; Baek et al. 2026). The therapeutic target is a defined acquisition or metabolic process whose manipulation improves the activity of a specified antibiotic without disabling host antimicrobial metabolism.

Accordingly, metabolic interventions should be evaluated across three experimentally separable outcomes: bacterial killing during antibiotic exposure, population recovery after exposure, and the relative fitness of susceptible and resistant genotypes. Benefit at one level alone is insufficient to predict treatment outcome.

Applied translation of the lineage model would require a measurable signature that identifies which carbon-conditioned survivors contribute to post-treatment population recovery. In diabetes-associated infection, systemic glycaemia is an accessible host variable, but it cannot be assumed to represent bacterial carbon exposure. Its prognostic or interventional value must first be linked to local substrate allocation, bacterial physiological state and lineage-resolved recovery. Such validation could ultimately support risk stratification and the design of metabolic co-interventions while distinguishing a mechanistically informative marker from a nonspecific correlate of metabolic comorbidity.

Resolving which intervention is appropriate requires experiments that preserve information currently separated across experimental systems: substrate identity, cellular acquisition, intracellular carbon fate, bacterial physiological state and cellular descent through time. Stable-isotope tracing can identify carbon fate, cell-resolved measurements can expose physiological heterogeneity, and longitudinal lineage approaches can determine whether cells surviving one metabolic state contribute disproportionately to populations recovered after environmental change. Coupling these measurements to antibiotic killing and post-regrowth susceptibility would distinguish metabolic states that merely coexist with treatment failure from those that causally determine which bacterial populations survive, recover and expand. Although the evidence reviewed here is organism-specific, the analytical distinction among extracellular pool, cellular access and intracellular fate is transferable to host–microbe systems in which multiple cell types compete for and transform the same nutrient. By separating causal levels and making cellular descent falsifiable, this framework defines the experiment needed to determine when carbon-conditioned survival becomes an evolutionary source of treatment failure.

## Data Availability

No datasets were generated or analysed during the current study.

## Declarations

### Use of generative AI

For the originally submitted manuscript, the corresponding author used ChatGPT (OpenAI) to check English grammar and spelling and to identify sentences whose wording could impede comprehension. The tool was not used to select or interpret studies, generate scientific claims, or draft sections of the original submission. The authors reviewed the suggested language changes and retained only those that preserved the intended scientific meaning. During revision, ChatGPT/Codex (OpenAI) assisted in identifying and summarizing publications and organizing the evidence matrix. The tools also proposed wording for additions on lineage tracking and on how the two-stage model relates to existing models of the transition from antibiotic tolerance to resistance. The tools also assisted with selected language and table edits. The authors evaluated the primary literature, determined which proposals to retain, revised the text, and take full responsibility for the scientific content and final manuscript.

Figure 1 was prepared by the authors in Microsoft PowerPoint using graphical elements available through Canva.
